# Whitening efficacy of popular natural products on dental enamel

**DOI:** 10.1590/0103-6440202204863

**Published:** 2022-06-24

**Authors:** Carla Roberta de Oliveira Maciel, Ayodele Alves Amorim, Rebeca Franco de Lima Oliveira, Rocio Geng Vivanco, Fernanda de Carvalho Panzeri Pires-de-Souza

**Affiliations:** 1 Department of Dental Materials and Prosthodontics, Ribeirão Preto School of Dentistry, University of São Paulo, Av do Cafe, s/nº, Ribeirão Preto, Sao Paulo, 14040-904, Brazil.

**Keywords:** Bleaching agents, natural products, toothbrushing, color stability, microhardness

## Abstract

The objective of this study was to evaluate the effect of natural bleaching products on the color, whiteness, and superficial properties of dental enamel. Seventy fragments of bovine teeth were obtained (6mm x 6mm x 2mm). Initial surface roughness (Surfcorder SE1700, Kosakalab), microhardness (HMV-2, Shimadzu), color (EasyShade, VITA), and surface gloss (Micro-Gloss 45º BYK, Gardner) readings were done. Samples were separated into five groups (n=14) according to the treatments used: CT-conventional toothpaste (negative control); CH-charcoal; TU-turmeric; BP-banana peel, and CP16%-16% carbamide peroxide gel (positive control, 4 h/day for 14 days), and then brushed for 560 cycles (T1) and 1200 cycles (T2), equivalent to 14 and 30 days of brushing. New measurements were performed after T1 and T2. The whiteness index for dentistry change (∆WID) and Weight loss (Wl) were calculated. CP16% demonstrated the highest (p<.05) color change (ΔE00) and ∆WID (2-way ANOVA, Bonferroni, p<.05). Surface gloss alterations were lower for TU, CP16%, and BP. CT and CH increased surface roughness (p<.05). CP16% decreased enamel microhardness. CH presented medium abrasiveness, and CT and TU, low abrasiveness. The popular bleaching products were not efficient for tooth whitening. Furthermore, brushing with charcoal increased the enamel surface roughness, and CP16% decreased enamel microhardness over time

## Resumo

O objetivo deste estudo foi avaliar o efeito de produtos clareadores naturais na cor, efeito clareador e propriedades superficiais do esmalte dentário. Setenta fragmentos de dente bovino foram obtidos (6mm x 6mm x 2mm). Foram realizadas leituras iniciais de rugosidade de superfície (Surfcorder SE1700, Kosakalab), microdureza (HMV-2, Shimadzu), cor (EasyShade, VITA) e brilho (Micro-Gloss 45º BYK, Gardner). As amostras foram separadas em cinco grupos (n=14), de acordo com os tratamentos utilizados: DC - dentifrício convencional (controle negativo); CA - carvão ativado; CM - cúrcuma; CB - casca de banana e PC16% - gel de peróxido de carbamida a 16% (controle positivo, 4h/dia por 14 dias), foram então escovadas por 560 ciclos (T1) e 1200 ciclos (T2), equivalente a 14 e 30 dias de escovação. Novas leituras foram realizadas após T1 e T2. A alteração do whiteness index for dentistry (∆WI_D_) e perda de massa (P_m_) foram calculadas. PC16% demonstrou a maior (p<0,05) alteração de cor (ΔE_00_) e ∆WI_D_ (2-way ANOVA, Bonferroni, p<0,05). A alteração de brilho foi menor para CM, PC16% e CB. DC e CA aumentaram a rugosidade de superfície (p<0,05). PC16% diminuiu a microdureza do esmalte. CA apresentou abrasividade média, e DC e CM, baixa abrasividade. Os produtos clareadores populares não foram eficientes para clareamento dental. Ademais, a escovação com carvão ativado aumentou a rugosidade de superfície do esmalte dentário, e com maior tempo PC16% diminuiu a microdureza do esmalte.

## Introduction

Nowadays, patients are increasingly demanding more from dental esthetics. Whitened teeth give people more social acceptance and satisfy their appearance ^1^. However, it is natural that teeth color changes over time. Oral hygiene, within this context, has the objective of maintaining dental esthetic through the removal of extrinsic stains caused by the acquired pigmented pellicle ^2^, which is related to the patients’ eating and hygiene habits, as well as their age. These factors, isolated or together, are related to the teeth color and surface alterations ^2^ ^,^ ^3^ ^,^ ^4^.

Tooth whitening products have a well-known action mechanism. Hydrogen peroxide oxidizes the chromogens’ double bonds, which become lighter colored. For the whitening gels based on the carbamide peroxide agent, carbamide peroxide breaks down in the presence of water, releasing the hydrogen peroxide ^5^. The 16% carbamide peroxide bleaching agent is the most common and the best seller “at home” bleaching agent. According to Llena et al. (2020) ^6^, treatment with 16% CP is an effective and safe tooth whitening procedure, and the color obtained remains stable over the long term. Nevertheless, the current treatment options seem to not fully satisfy the increasing need for whiter teeth ^4^.

Because of the current unachievable beauty standards and social pressure that are broadcasted through social media, every day, new videos and profiles emerge from the internet recommending the use of homemade products, to obtain tooth whitening, with the promise of fast and cheap dental bleaching, without any scientific evidence about their efficacy and safety to the oral health ^7^ ^,^ ^8^. We have activated charcoal, turmeric, and banana peel within the most recommended natural products.

Currently, activated charcoal is one of the most popular and appealing products. It is being commercialized as an oral hygiene product due to its adsorption capacity for pigments responsible for tooth color change ^9^. Although manufacturers assure whitening, remineralization, and antimicrobial activity, there is insufficient scientific evidence to support these promises ^10^.

Curcumin is the most bioactive component of turmeric, and it is known for its antioxidant, antibacterial, and anti-inflammatory activities. Besides, curcumin presents low water solubility, low chemical stability, and oral biodisponibility ^11^ ^,^ ^12^. In dentistry, its therapeutical effects have been studied on neoplasms and oral mucous affections as an antimicrobial agent ^12^ ^,^ ^13^.

Banana peels are commonly considered as residue. However, they present in their composition important compounds, such as flavonoids, alkaloids, tannins, quinones, and saponins, which have antioxidants and anti-inflammatory properties ^14^ ^,^ ^15^. Banana peel extracts also demonstrate antimicrobial activity against pathogens that cause oral diseases ^16^.

Therefore, the aim of the study was to analyze the effect of brushing with popular natural agents, used by the population to obtain tooth whitening but not indicated for that purpose, on the color, whitening, and superficial properties of dental enamel. The null hypothesis was that there would be no difference in the dental enamel brushed with natural substances compared to the conventional toothpaste regarding the color change, surface gloss, surface roughness, and microhardness.

## Materials and methods

### Sample selection

The sample size (n=14) was based on data obtained from a pilot study and determined on www.openepi.com, with a confidence interval of 95% and power of 80%. For this study, sound bovine teeth without cracks or fractures were used. After removing the roots, the crowns were cut using a low-speed diamond saw (Isomet 1000, Isomet, Buehler, Lake Bluff, IL, USA) underwater cooling to obtain 70 fragments (6 mm x 6 mm x 2 mm).

The enamel surface was polished using abrasive papers under refrigeration (600, 1200, and 2000 grits) so that the surface roughness would not exceed 0,4 µm. Then, the samples were separated and stored in artificial saliva at 37 ºC.

### Sample readings

The surface roughness, microhardness, color, and surface gloss readings were done at 3 different times: before the treatments (T0), after 560 brushing cycles (T1), and after 1200 brushing cycles (T2).

### Surface roughness

The initial surface roughness readings (Surfcorder SE 1700, Kosakalab, Tokyo, Japan) were done. Three readings were performed on the enamel surface of each sample: in the middle, 1 mm to the right, and 1 mm to the left. The mean value of the readings was used as the initial surface roughness value. To calculate the surface roughness alteration, the following formula was employed:

∆Ra=Ra_f-Ra_i

Where Rɑ_i_ is the initial surface roughness value and Rɑ_f_ the final one. ΔRɑ was calculated after T1 and T2 (Rɑ_f_).

### Microhardness analysis

For the Knoop microhardness analysis (Micro Hardness Tester HMV-2, Shimadzu®, Tokyo, Japan), a statical vertical load of 25 g was applied for 5 seconds ^17^. Like the surface roughness readings, three initial readings were done: in the middle, 1 mm to the right, and 1 mm to the left. The mean of the three readings was considered as the initial microhardness value. To calculate the relative microhardness, the following formula was used:

$$∆KHN_{r\;}=(KHN_{f}/KHN_{i})\;x\;100\;$$

Where KHN_i_ is the initial microhardness value and KHN_f_ the final one. ΔKHN_r_ was calculated after T1 and T2 (KHN_f_) and presented as percentages (%).

### Color analysis

For the color readings, a spectrophotometer was employed (EasyShade, VITA Zahnfabrik, Bad Sckingen, Germany). The samples were placed over a white background (White Standard Sphere for 45º, 0º Reflectance and Color Gardner Laboratory Inc. Bethesda, Geretsried, Germany) in a standardized lightbox (Gester International, Fujian Province, China). The standard illuminant used was D65, which simulates the daylight spectrum.

Three color readings were performed for each sample using the CIE L* a* b* coordinates, and the mean of the three readings was considered the color value on the L*, a*, and b* axis. For standardization purposes, samples with values of L*, a*, and b* ranging over 0.5 in each coordinate were discarded.

The color stability was calculated using the L*, a*, and b* initial and final values, with the CIEDE2000 formula ^16^ ^,^ ^17^ ^,^ ^18^ ^,^ ^19^:

$${∆E}_{00\;\;}={\;\left[{\left(\frac{{∆L}^{'}}{{\;K}_{L\;}S_{L\;\;}\;\;}\right)}^{2}+\;{\left(\frac{{∆C}^{'}}{\;K_{C\;}S_{C\;\;}\;\;}\right)}^{2}+{\left(\frac{{∆H}^{'}}{{\;K}_{H}S_{H\;\;}\;\;}\right)}^{2}+R_{T}\;\left(\frac{{∆C}^{'}}{{\;K}_{C\;}S_{C\;\;}\;\;}\right)\left(\frac{{∆H}^{'}}{{\;K}_{H}S_{H\;\;}\;\;}\right)\;\right]}^{0,5}$$

Where ΔL’, ΔC’, and ΔH’ are the differences in lightness, chroma, and hue, respectively, between two measures and RT (rotation function) is a function that accounts for the interaction between chroma and hue differences in the blue region. S_L_, S_C_, and S_H_ are the weighting functions for the lightness, chroma, and hue components, respectively; and K_L_, K_C_ and K_H_, the parametric factors according to different viewing parameters set to 1 ^18^ ^,^ ^19^.

The color variation values were compared to the perceptibility (0,8) and acceptability ^1^ ^,^ ^8^ thresholds ^20^.

### Whiteness Index for Dentistry (WI_D_)

The whitening index for dentistry (WI_D_) analysis correlates the whitening visual perception, avoiding bias from the subjective visual factor on the dental color evaluation. It is calculated using the L* a* and b* coordinates, with the following formula:

$$WI_{D}=0.511L^{*}-2.324a^{*}-1.100b^{*}$$

The WI_D_ was determined after each tested time. Whiteness differences (∆WI_D_) were calculated after T1 and T2 in relation to baseline values. CIELAB values close to reference white (L* = 100, a* = 0, b* = 0) indicate higher whiteness value. So, positive ∆WI_D_ values indicate higher whitening perceptibility, and lower (or negative) ones indicate lower whitening perceptibility. The ∆WI_D_ values were compared to the perceptibility (0,72) and acceptability (2,60) thresholds ^21^.

### Surface Gloss Analysis

For the surface gloss analysis, a glossmeter was used (Micro-Gloss 45º, BYK Gardner, Geretsried, Germany), with 45º geometry reading ^22^. The light is reflected the surface of the enamel at a defined angle and measured in numerical values. The values can vary from 0 to 100 GU (gloss unit).

Three readings were performed for each sample, and the mean of the readings was considered the gloss value. The gloss alteration was calculated by the final and initial values difference for each time (T1 and T2), using the following formula:

$$∆GU=GUt_{x\;}-GUt_{0}$$

GUt_0_ is the initial gloss value and GUt_x_ is the value after T1 and T2.

### Simulated brushing

The samples were separated into five groups (n = 14) according to the treatment used (conventional toothpaste, activated charcoal, turmeric, banana peel, and CP16%, Box 1. The simulated brushing was performed in a simulating toothbrushing machine (Pepsodent, MAVTEC - Com. Peças, Acess. e Serv. Ltda. ME, Ribeirão Preto, SP, Brazil) using toothbrushes with soft bristle (Johnson & Johnson Ind. Com. Ltda., São José dos Campos, SP, Brazil) for each sample, according to ISO/DTS 145692 50. The toothbrushing machine was set to 356 rpm with a 200 g load. At T1, 560 cycles were done, and at T2, 1200 cycles, simulating 14 and 30 days of brushing by a healthy individual, respectively ^23^.

The samples brushed with the banana peel were positioned in a personalized rectangular acrylic resin appliance which allowed the samples to be rubbed against the banana peel. The banana peels were cut 55 cm in length and positioned in the machine. According to the previous pilot study, the peels were replaced every 30 seconds due to the fast deterioration. This test simulated the rubbing of the peels based on the same strength used in manual toothbrushing.

**Box 1:**
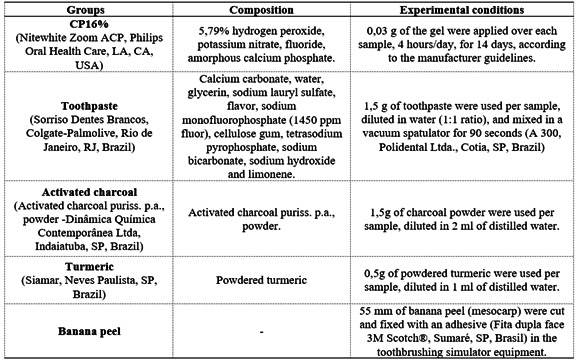
Groups distribution, products and materials used for each group and treatment methods.

The CP16% group (positive control) was treated using the 16% carbamide peroxide gel (Nitewhite Zoom, Philips Oral Health Care, LA, CA, USA) ^24^. The samples were positioned on a flat surface, and 0,03 g of the gel was applied over the enamel surface for 4 hours/day, for 14 days, according to the manufacturer’s guidelines.

After treatments, the samples were rinsed with distilled water for 10 seconds, immersed in artificial saliva, and stored at 37 ºC.

### Weight loss

The weight loss test was performed to measure the abrasiveness of the products used. For that 18 plexiglass samples (Riberman Plásticos Industriais Ltda., Ribeirão Preto, SP, Brazil) with 90 mm x 30 mm x 4 mm were used. The plexiglass samples were immersed in distilled water and stored at 37 ºC. After one month, three weightings were performed for each sample, and the mean was used as the initial mass value. Then, the samples were randomly separated into three groups (n = 6) according to the product used for the simulated brushing. The plexiglass samples were brushed for 41 minutes (14600 = 1 year of brushing by a healthy individual) ^24^. After that, the final weightings were done. The initial and final mass values were used to calculate the weight loss (Wl) using the following formula:

Wl=wF- wI

Where wI is the initial mass value and wF the final one. Weight loss values under 21 mg indicate low abrasiveness; between 21 and 40 mg, medium abrasiveness and over 41 mg, high abrasiveness ^25^.

### Scanning electron microscopy - SEM

Initially, two bovine teeth samples were obtained and polished as a control, and two of each group were randomly selected after treatments. The surface morphology of the enamel was analyzed through scanning electron microscopy (SEM, EVO MA10, ZEISS). For that, the samples were desiccated for 12 hours using a desiccator with silica gel. Then, the samples were placed in aluminum stubs (Electron Microscopy Sciences, Washington, EUA), sputter-coated with gold-palladium alloy (Bal-Tec, model SCD 050 sputter coater, Balzers, Liechtenstein), and observed at 200x and 1000x magnifications (20 kV, 30 mm WD and spot size 28 mm) ^26^.

### Statistical analysis

The data were submitted to the Shapiro-Wilk (p < .05) normality test and analyzed by 2-way ANOVA (variation factors: treatments and time), with repeated measures, and by Bonferroni’s test (p < .05), with the exception of the weight loss (Wl), which was analyzed through one-way ANOVA and Tukey’s test (p < .05).

## Results

**Figure 1:**
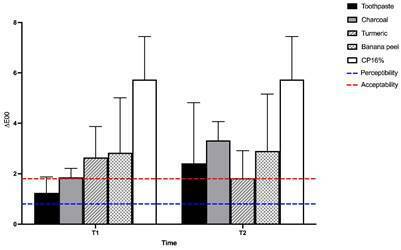
Means comparison of (E_00_ (2-way ANOVA, Bonferroni’s Test, p < .05), and limits of perceptibility (0.8) and acceptability (1.8).

Regarding color change, all treatments caused changes in (E_00_ (Figure 1). However, only the CP16% group presented a significant difference (p < .05), irrespective of the brushing times. The samples treated with conventional toothpaste and charcoal showed color variation within the acceptability threshold ^20^ after 14 days of brushing. After 30 days, only turmeric resulted in color variation within the acceptability threshold.

**Figure 2:**
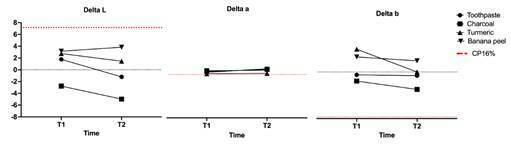
Alteration in ∆L, ∆a, and ∆b between the time of use (T1 and T2) for each experimental group.

When analyzing the (L*, (a*e (b* mean values (Figure 2), after treatments, it is noticed a decrease in the lightness of the samples brushed with charcoal for both 14 and 30 days. Regarding (a*, the variation was slightly similar for all groups, regardless of the brushing time. There was an increase for (b* after 14 days, demonstrating a yellowing for the samples treated with turmeric and banana peel. However, after 30 days of brushing with turmeric, there was a decrease in (b*. The highest decrease in the (b* was observed in the samples brushed with activated charcoal after the 30 days period.

**Figure 3:**
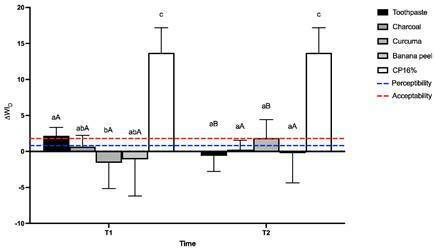
Means comparison of ∆WI_D_ (2-way ANOVA, Bonferroni’s Test, p < .05). Different letters, lowercase on the same time (T1 or T2) and uppercase between the time of use (T1 x T2), indicate a statistically significant difference (p < .05).

Concerning the ∆WI_D_ (Figure 3), the highest whitening values were found in the samples treated with CP16%, different (p<.05) from all the other groups. When comparing the ∆WI_D_ to the acceptability and perceptibility thresholds, it was found that after 14 days, the conventional toothpaste and CP16% groups presented values above both thresholds. After 30 days, the turmeric and CP16% groups also showed values above both thresholds. Charcoal and banana peel groups could not reach the whitening perceptibility threshold, regardless of the time of use. The conventional toothpaste surpassed the acceptability whitening threshold after 14 days of brushing, and the same happened for the turmeric group.

The treatment with turmeric, banana peel, and CP16% reduced the enamel surface gloss (Table 1); and the treatment with charcoal, after 30 days, resulted in the highest enamel surface gloss, different (p > .05) from all the other groups, except for the conventional toothpaste (p > .05).

**Table 1:** Means and standard deviation of surface gloss, microhardness (%), and surface roughness of the experimental groups (2-way ANOVA, Bonferroni test, p < .05).

		Toothpaste	Charcoal	Turmeric	Banana peel	CP16%
(GU	T1	0,06 (1,22) aA	1,04 (1,73) aA	-4,37 (2,63) bA	-3,24 (2,22) bA	-3,83 (3,22) b
	T2	0,16 (1,11) acA	1,71 (2,38) cA	-2,96 (2,64) abA	-2,84 (6,3) abA	
ΔKHN_r_	T1	46,58 (53,31) aA	37,31 (21,66) acA	5,47 (27,94) bA	8,32 (18,93) bcA	-14,46 (23,39) b
	T2	55,52 (30,50) aA	53,06 (30,72) aA	2,85 (23,94) bA	0,44 (25,60) bA	
Ra	Baseline	0,12 (0,02) aA	0,12 (0,04) aA	0,13 (0,04) aA	0,14 (0,06) aA	0,15 (0,06) aA
	T1	0,44 (0,13) aB^*#*^	0,32 (0,11) bB^*#*^	0,17 (0,06) cA	0,14 (0,09) cA	0,15 (0,09) cA
	T2	0,38 (0,18) aB^*#*^	0,42 (0,25) aC^*#*^	0,13 (0,06) bA	0,17 (0,10) bA	

Different letters, lowercase on the line and uppercase on the column, indicate statistically significant difference (p < .05).

When analyzing the microhardness values (Table 1), CP16% decreased the enamel microhardness. The banana peel and turmeric groups presented similar values (p < .05) with a little increase in microhardness, regardless of the time of use. Brushing with conventional toothpaste and charcoal resulted in higher relative microhardness, different from the other groups (p < .05) and similar to each other (p > .05).

On the other hand, brushing with activated charcoal resulted in higher surface roughness values (p < .05) throughout 30 days of treatment (Table 1). The turmeric and banana peel treatments did not present any significant difference (p > .05) in the enamel surface roughness.

The results obtained after the abrasiveness test (Table 2) showed that brushing with turmeric presented less weight loss values when compared to all the other treatments (p < .05). There was no difference (p > .05) in the conventional toothpaste and charcoal weight loss. Nevertheless, the charcoal abrasiveness resulted in a weight loss of over 21 mg, indicating medium abrasiveness according to ISO 8627 ^27^, while the conventional toothpaste resulted in low abrasiveness (< 21 mg of substance loss).

**Table 2:** Mean and standard deviation for weight loss (mg) for the experimental groups (One-way ANOVA, Turkey’s test, p < .05).

Toothpaste	Charcoal	Turmeric
13.89 (3.28) A	22.67 (10.24) A	3.33 (5.16) B

Different uppercase letters on the line indicate statistically significant (p < .05).

**Figure 4:**
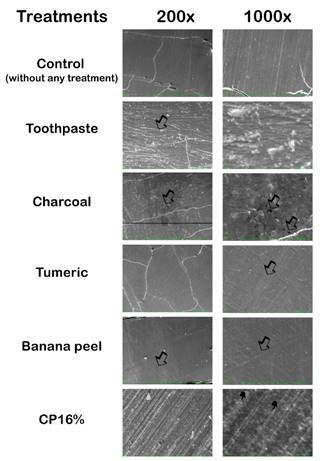
Representative photomicrographs of the images were obtained with SEM. Arrows represent morphological alterations on dental enamel, and finger points show enamel prisms.

SEM images were obtained from the samples treated and not treated (used as control), allowing the comparison between them (Figure 4). In the control samples, it is possible to observe the homogenous polish of the enamel surface (Figure 4). For the brushed samples, wear marks on the enamel surface were observed (arrows), being more pronounced on the samples brushed with charcoal, clearly visualized on the 1000x magnification (Figure 4).

The enamel surface treated with CP16% showed at the 200x magnification interprismatic among prismatic enamel, all over the surface with a uniform distribution (Figure 4). SEM image with 1000x magnification revealed some enamel prisms' protrusion and elongated shape (Figure 4), presenting a rough surface appearance.

## Discussion

The aim of this study was to evaluate the effect of brushing with popular natural agents, used by the population to obtain tooth whitening but not indicated for that purpose, such as turmeric, charcoal, and banana peel, widely spread on the internet without any scientific evidence. The null hypothesis was that there would be no difference in the color, whitening and superficial properties of the dental enamel brushed with those products, in comparison to a conventional toothpaste, as a negative control. A positive control group was also included in this study, with 16% carbamide peroxide gel, since its efficacy is already known in the literature, to compare the potential bleaching effect of these natural products with a well-established whitening agent. The null hypothesis was rejected because the bleaching agents altered all the properties tested, except for the color.

Regarding color variation, there was no significant difference (p > .05) in (E_00_ of the samples treated with the natural products compared to conventional toothpaste, regardless of the brushing time. The CP16% presented higher color change, different (p < .05) from all the other groups, as demonstrated in other studies ^1^ ^,^ ^28^ ^,^ ^29^.

Despite the lack of significant differences among the natural agents, all treatments resulted in color change above the perceptibility threshold ((E_00_ > 0,8), regardless of the time of use, results similar to the ones found by Franco et al. (2020) ^10^. Nevertheless, the conventional toothpaste and charcoal presented color variation below the acceptability threshold after 14 days, similar to the effect caused by turmeric after 30 days of brushing. All the other treatments caused clinically unacceptable color change ((E_00_ > 1,8) irrespective of the brushing time.

The comparison between the mean values of (L*, (a* e (b*, after T1 and T2, enables the analysis of these variations, as established in the CIE L*a*b* color space. Thus, the initial (L* variation demonstrated that the charcoal caused a darkening of the samples after 14 days of brushing, and these results did not change after 30 days. Turmeric and banana peel caused positive (L* variation, and the conventional toothpaste initially caused a positive (L* change, but after 30 days, the samples were darkened.

The (a* variation was slight and similar for all groups, regardless of the time of use, indicating no change in the red/green axis. The initial (b* variation demonstrated that the charcoal caused a decrease in the yellow chroma, being more pronounced after 30 days. On the other hand, when treated with turmeric and banana peel, it resulted in a yellowing of the samples. After 30 days, the samples brushed with turmeric presented a decrease in the yellow saturation, while the banana peel maintained the initial value of (b*.

The low ∆a* variation is expected once the highest whitening alterations occur on the other coordinates ^30^. The increase in the yellow chroma of the samples brushed with turmeric can be justified by essential oils in these rhizomes in natura ^31^. The decrease of the yellow chroma after 30 days can be explained by the fact that turmeric is a non-polar polyphenol ^32^. Pigmented solutions such as coffee and tea with high polarity leach out, causing color change through the pigmentation of enamel chromophores ^33^. Since turmeric is non-polar, there is no leaching, resulting in a lower penetration capacity. Therefore, when brushing for a longer period, equivalent to 30 days, the turmeric abrasiveness could remove the initial staining (after 14 days), which would justify the ∆b* results after 30 days. Similar results were found by El Bishbishy et al. (2021) ^34^, where they evaluated the color of toothpaste based on turmeric extract.

 The increase in the yellow saturation caused by the friction of banana peel can be explained by the incorporation of carotenoid pigments from the peel on the enamel surface ^35^. Besides, only ripe banana peels were used, which present elevated compound levels, increasing its staining potential ^16^ ^,^ ^35^.

The bleaching effect of charcoal is based on the adsorption of pigments ^36^. The activated charcoal binds to the tooth surface, to the chromophores and pigments, acting as a filter for these staining agents, presenting, in theory, the potential to alter the tooth color ^9^. However, many factors interfere with its abrasiveness, such as the way of obtaining the charcoal, its composition, and the particles size ^37^.

The WI_D_ is the whitening index for dentistry, and it is used to evaluate the whitening perceptibility, correlating the visual assessments to the CIE L*a*b* coordinates. The application of this index decreases the subjectiveness of the visual analysis and quantifies the whitening effect. High positive WI_D_ values indicate higher whitening perceptibility, while low values are related to lower whitening perceptibility ^38^,^39^.

The results found for the ∆WI_D_ reject the null hypothesis because the whitening index of the turmeric group was different from the conventional toothpaste after 14 days of brushing, resulting in negative values. Charcoal and banana peel did not achieve the perceptibility threshold, regardless of the time of brushing, demonstrating that these products did not change the whitening perception of the samples.

Negative ∆WI_D_ values are observed in samples submitted to staining protocols ^36^. Therefore, brushing with turmeric for 14 days presented a staining ability, resulting in a negative whitening index, different (p < .05) from the samples brushed with the conventional toothpaste. Turmeric is considered a natural pigment due to curcumin ^40^. Turmeric roots also present a high concentration of essential oils that contribute to pigmentation ^32^. Therefore, when settled, the pigment does not improve the whitening perception expressed by the ∆WID ^38^. A similar activity occurs due to banana peel with the carotenoids ^41^.

The activated charcoal is highly porous, and the whitening effect of the product is based on its capacity to adsorb and retain chromophores of the diet in the oral cavity. Despite this, there was a slight positive change for the charcoal group for the WI_D_, demonstrating that the use of charcoal does not influence the whitening perception of the dental enamel. Our results corroborate Brooks et al. (2017) study ^42^, according to whom there would be no free radical bleaching agent available in charcoal, reducing the capacity of intrinsic staining alteration in enamel.

The tooth surface morphology affects the quantity and type of light reflected. A rougher surface allows higher diffuse reflection, while a flat surface results in specular reflection. The amount of light reflected over the enamel surface after brushing increased. Thereby, surface gloss can alter color perception ^43^. In that way, in the present study, the gloss alteration can be justified by increased surface roughness. Any surface irregularity can alter the direction of the light reflected, resulting in different quantities of light reflected in the sensor, compromising the results ^44^.

Changes in the enamel surface roughness interfere with the perception of color and surface gloss because those alterations can lead to scattering and diffuse light reflection ^45^. The samples brushed with the conventional toothpaste and charcoal had higher surface roughness after 14 days of treatment (p < .05), and for the charcoal, this increase was even higher after 30 days. The other products did not present a significant difference (p > .05) related to the initial values. So, the activated charcoal presented the highest surface roughness values due to its abrasiveness.

The samples brushed with the conventional toothpaste and charcoal revealed higher surface roughness values. They increased the surface gloss, probably the abrasiveness of the products may have caused alterations on the enamel surface that changed the reflection of the light ^46^.

The relative microhardness expresses the increase or decrease of the enamel microhardness after treatments, related to the initial values. Negative relative microhardness values demonstrate a decrease in the final microhardness, and positive values indicate an increase in the microhardness. So, analyzing the results found there was no significant difference (p > .05) between T1 and T2 for all the treatments. Only CP16% decreased the microhardness of the enamel, similar (p > .05) to the turmeric and banana peel groups but different (p<.05) from the conventional toothpaste and charcoal groups.

Harrington et al. (1982) ^47^ adapted the weight loss method from Epstein and Tainter (1943) ^48^, which measured the abrasiveness of the toothpaste on metal plaques, to be used on acrylic ones, which according to the authors, present the same hardness as the human dentin. To determine the abrasiveness of toothpaste, the weight loss method is used, ranging the products as low, medium, and high abrasiveness. According to ISO 8627 ^27^, the product is low-abrasive when presenting a weight loss value under 21 mg; medium-abrasive, between 21 and 40 mg; and high-abrasive, over 41 mg. So, there is a direct relation between toothpaste abrasiveness and weight loss, in a way that higher weight loss, higher toothpaste abrasiveness. Hence, according to the results found, conventional toothpaste presents low abrasiveness; charcoal, medium abrasiveness; and turmeric, low abrasiveness. Weight loss analysis was not performed with the banana peel group because of its fast degradation in the simulating toothbrushing machine. Also, the friction over the acrylic slide would not efficiently assay the abrasiveness.

When associating the weight loss and surface roughness results, rougher enamel surfaces for charcoal were found, followed by conventional toothpaste, corroborating the results found by Palandi et al. (2020) ^49^. According to the authors, longer toothbrushing with more abrasive agents can decrease the dental enamel volume ^49^. Turmeric was the least abrasive agent with lower enamel surface roughness alteration, which can be justified by the presence of essential oils on the turmeric composition ^31^. When rubbed, turmeric releases its oils, decreasing the abrasiveness, resulting in a lower weight loss and lower surface roughness.

The SEM analysis corroborates the surface roughness, microhardness, and weight loss results. The images obtained as control showed a smooth enamel surface with some scratches. These irregularities may have resulted from the polishing process, necessary for the standardization of the enamel surface. Considering that a rough surface contributes to enamel staining ^50^, all the samples were flattened and polished until the surface roughness achieved 0,4 µm. So, after the treatments, rougher surfaces indicate that the treatments altered the samples.

According to Silva et al. (2018) ^51^, some alterations in the enamel surface are more pronounced after brushing with toothpaste, justified by its abrasiveness. Results in accordance with our findings. The activated charcoal also produced deep wear marks, probably due to its abrasiveness. Thus, conventional toothpaste and charcoal use evidenced the enamel irregularities already present or caused by the polishing process. Brushing with turmeric and banana peel caused minor alteration on the enamel surface. As previously cited, the release of essential oils may decrease the abrasiveness of the products, resulting in less alteration.

Based on the results found, it was concluded that the popular natural agents used to obtain tooth bleaching but not indicated with that purpose did not present whitening efficacy, regardless of the time of use. Changes in the surface gloss of the enamel are related to alterations in the surface roughness of this substrate. The proposed bleaching agents can alter the enamel surface roughness. The conventional toothpaste, charcoal, and carbamide peroxide gel caused alteration in the enamel surface, different from turmeric and banana peel.
